# Bacterial Subversion of Autophagy in Cystic Fibrosis

**DOI:** 10.3389/fcimb.2021.760922

**Published:** 2021-10-08

**Authors:** Verónica Roxana Flores-Vega, Silvia Yalid Vargas-Roldán, José Luis Lezana-Fernández, Ricardo Lascurain, José Ignacio Santos-Preciado, Roberto Rosales-Reyes

**Affiliations:** ^1^ Unidad de Medicina Experimental, Facultad de Medicina, Universidad Nacional Autónoma de México, Mexico City, Mexico; ^2^ Escuela de Ciencias de la Salud, Universidad del Valle de México, Campus Coyoacán, Mexico City, Mexico; ^3^ Departamento de Microbiología, Escuela Nacional de Ciencias Biológicas del Instituto Politécnico Nacional, Mexico City, Mexico; ^4^ Laboratorio de Fisiología Respiratoria y la Clínica de Fibrosis Quística, Hospital Infantil de México Federico Gómez, Mexico City, Mexico; ^5^ Dirección Médica, Asociación Mexicana de Fibrosis Quística, Mexico City, Mexico; ^6^ Departamento de Bioquímica, Facultad de Medicina, Universidad Nacional Autónoma de México, Mexico City, Mexico

**Keywords:** cystic fibrosis, autophagy, subversion, *Burkholderia cenocepacia*, *Pseudomonas aeruginosa*

## Abstract

Cystic fibrosis (CF) is a genetic disease affecting more than 70,000 people worldwide. It is caused by a mutation in the *cftr* gene, a chloride ion transporter localized in the plasma membrane of lung epithelial cells and other organs. The loss of CFTR function alters chloride, bicarbonate, and water transport through the plasma membrane, promoting the production of a thick and sticky mucus in which bacteria including *Pseudomonas aeruginosa* and *Burkholderia cenocepacia* can produce chronic infections that eventually decrease the lung function and increase the risk of mortality. Autophagy is a well-conserved lysosomal degradation pathway that mediates pathogen clearance and plays an important role in the control of bacterial infections. In this mini-review, we describe the principal strategies used by *P. aeruginosa* and *B. cenocepacia* to survive and avoid microbicidal mechanisms within the autophagic pathway leading to the establishment of chronic inflammatory immune responses that gradually compromise the lung function and the life of CF patients.

## Introduction

Autophagy is a self-degradative process that plays a key housekeeping role in removing misfolded or aggregated proteins located in the cytosol. This cellular process contributes to the removal of damaged organelles including mitochondria, peroxisomes, and endoplasmic reticulum (Dikic and Elazar, 2018). Autophagy also plays an important role in the regulation of inflammasome activation, particularly in the removal of inflammasome-activating endogenous signals as well in the sequestration and remotion of inflammasome components (Harris et al., 2017). In innate immunity, autophagy plays a role in controlling the intracellular spread of cytosolic bacteria and restricting bacteria contained in vacuoles or phagosomes. During bacterial infection, infected cells form double-membrane compartments (known as autophagosomes) around free bacteria or associated to damaged vacuoles by intracellular pathogens that usually are delivered to lysosomes for their degradation (Huang and Brumell, 2014). As a cellular process, autophagy is highly efficient; nevertheless, some intracellular bacteria have evolved different strategies to avoid its degradation by the autophagic pathway.

## Pathophysiology of Opportunistic Infections in Patients With CF

Cystic fibrosis (CF) is an autosomal recessive congenital disease (O’Sullivan and Freedman, 2009; Shteinberg et al., 2021) that principally affects lungs, pancreas, liver, kidneys, and intestine of at least 70,000 people worldwide (Jackson and Goss, 2018). The condition is due to a mutation in the *cftr* gene (Tsui et al., 1985), that codes a CF transmembrane conductance regulator (CFTR) involved in the transport of chloride and sodium ions, $\text{HC}{\text{O}}_{3}^{-}$ , and water across the lung epithelia (Shteinberg et al., 2021). Defective CFTR function produces a thick and sticky mucus (Boyle, 2007) that rapidly clogs the lower airways in which diverse bacterial pathogens might produce infection and inflammation that gradually decrease the lung function (Blanchard and Waters, 2019), leading to the production of thick sticky mucus. Affected individuals develop shortness of breath, cough, and chronic infections that eventually decrease lung function, which increases the mortality risk. Several mutations are described in the *cftr* gene (Bareil and Bergougnoux, 2020). The most common mutation is the deletion of phenylalanine in the position 508 (F508del). This mutation is associated with inflammation and decreased autophagy (Luciani et al., 2010). The first bacterial pathogens associated with the lower airways of children with CF are non-typable *Haemophilus influenzae* and *Staphylococcus aureus* (Cox et al., 2010). This initial colonization is progressively replaced by *Pseudomonas aeruginosa* and *Burkholderia cenocepacia* during adolescence and adulthood. Both of the latter opportunistic pathogens produce chronic infections that gradually reduce the lung function (Cox et al., 2010; Rossi et al., 2020; Rosales-Reyes et al., 2021). The inefficient bacterial clearance by affected individuals with CF is associated with a reduced bactericidal activity of macrophages, neutrophils, and respiratory epithelial cells (Yoshimura et al., 1991; Smith et al., 1996; Painter et al., 2006; Porto et al., 2011). In the mouse model, the phagocytic activity of alveolar macrophages with a deficiency in CFTR (*cftr* ^-/-^) is not affected; however, its lysosomes fail to acidify and kill internalized bacteria (Di et al., 2006). *B. cenocepacia* invades macrophages and resides in a vacuole (BcCV) that shows a delay in lysosomal fusion (Lamothe et al., 2007). The delay in the lysosomal fusion with the BcCV is more pronounced in macrophages defective in CFTR (Lamothe and Valvano, 2008). In addition, *P. aeruginosa* survives more efficiently in macrophages with defective CFTR function (Porto et al., 2011) due to a deficiency in its lysosomal acidification (Di et al., 2006). In this mini-review, we describe how the subversion of autophagy by two important bacterial pathogens, *B. cenocepacia* and *P. aeruginosa*, contributes to the establishment of chronic infections in individuals with CF.

## Autophagy in CF

Autophagy is a cellular process that plays an important role in innate immunity, specifically by restricting the replication of bacterial pathogens contained in vacuoles or phagosomes. In CF, phagocytic cells increase the production of reactive oxygen species (ROS). The cells also increase the activation of the transglutaminase-2 (TGM2) that inactivates the Beclin1 (BECN1) complex resulting in an inefficient autophagy process (Luciani et al., 2010). Beclin1 is cross-linked by TGM2, and this new complex is sequestered in the cytosol to form aggresomes. The treatment of cells that express CFTR-F508del with Cysteamine corrects the autophagy deficiency by increasing the function of the BECN1 complex with a reduced level of sequestosome 1 (SQSTM1, also known as p62) (De Stefano et al., 2014; Ferrari et al., 2017). Cells with deficient autophagy in the airways of CF patients show accumulation of SQSTM1, a protein that works as an adaptor in the regulation of the formation and elimination of aggregates containing ubiquitinated proteins (Komatsu et al., 2007; Nezis et al., 2008). In addition, the accumulation of SQSTM1 at the endosomal level reduces the pool of the small GTPases Rab5 (Villella et al., 2013) and Rab7 (Gilardini Montani et al., 2019) that are essential for maturation to early and late endosomes, respectively. In addition, the dendrimer-based cysteamine analogue (PAMAM-DEN^CYS^) partially rescues the function of cells carrying the F508del mutation. This analogue significantly reduces the aggresome bodies formation in IB3 cells (Brockman et al., 2017). Thus, the autophagy dysfunction could be exploited by intracellular pathogens such as *B. cenocepacia* or *P. aeruginosa* to survive and persist in eukaryotic cells (Porto et al., 2011; Assani et al., 2014).

## Burkholderia cenocepacia

*B. cenocepacia* is a nonfermenting, anaerobic Gram-negative bacterium that belongs to the *Burkholderia cepacia* complex (Bcc) (Mahenthiralingam et al., 2005). *B. cenocepacia* and *B. multivorans* are two opportunistic pathogens that cause infections in individuals with CF. *B. cenocepacia* produces a chronic infection that is characterized by the establishment of a strong inflammatory immune response and cell death (Kopp et al., 2012). This bacterial infection decreases lung function (Scoffone et al., 2017) and reduces the survival of colonized individuals (Isles et al., 1984; Tablan et al., 1985).

**Figure 1:**
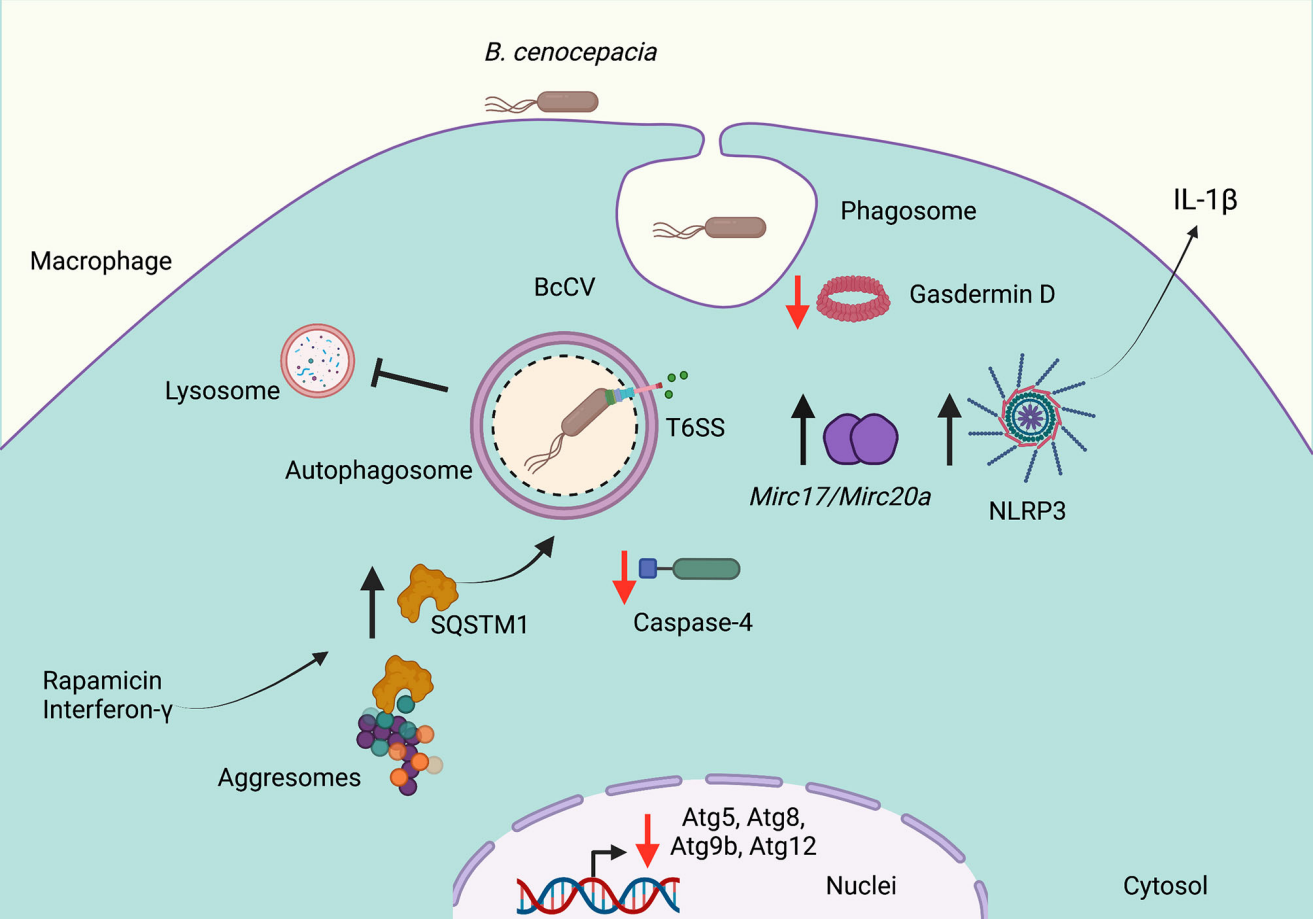
*Burkholderia cenocepacia* subverts autophagy in cystic fibrosis. *Burkholderia cenocepacia* invades phagocytic cells and resides in a vacuole (BcCV) that shows delayed fusion with lysosomes. The activity of the Type VI Secretion System (SST6) damages the membrane of the BcCV, allowing leakage of its content to activate the inflammasome NLRP3 and to release IL-1β. The damaged membrane of the BcCV might be surrounded by autophagosome membranes. Bacterial infections decrease the *Atg9b, Atg5, Atg12*, and *Atg8* transcription. The decrease of Gasdermin D and caspase-4 expression in *B. cenocepacia*-infected macrophages decreases the autophagosome formation. The downregulation of *Mir17* and *Mir20a* partially restored the autophagy deficiency. Rapamycin or IFN-γ stimulation induces the release of SQSTM1 from aggresomes to increase the mature autophagosome formation. Red arrows indicate decreased autophagy, and black arrows indicate increased autophagy. Created with BioRender.com.

*B. cenocepacia* invades macrophages and epithelial cells in which it persists and replicates (Burns et al., 1996; Saini et al., 1999; Martin and Mohr, 2000). In CF epithelial cells, *B. cenocepacia* resides in autophagosomes that fail to fuse with lysosomes (Sajjan et al., 2006). In macrophages, *B. cenocepacia* survives in a membrane-bound vacuole (BcCV) (Burns et al., 1996; Martin and Mohr, 2000) in which the bacteria delays the lysosomal fusion with the BcCV (Lamothe et al., 2007). *B. cenocepacia* also modulates macrophage function through the translocation of bacterial effectors by their type VI secretion system (T6SS) to inactivate the small GTPase Rac1 and decrease the ROS production (Rosales-Reyes et al., 2012b). During this process, the overexpression of the T6SS damages the membrane of the BcCV allowing leakage of its content to activate the inflammasome NLRP3 (Rosales-Reyes et al., 2012a) (**Figure 1**). The damaged membrane of the BcCV could be a signal to induce autophagy; however, *B. cenocepacia* impairs the formation of mature autophagosomes. The deficiency of caspase-4 (CASP-4, a protein associated to non-canonical activation of inflammasome) increases bacterial replication, with reduced association of LC3 at the BcCV. These observations suggest that CASP-4 has an important role in the autophagosome formation to control intracellular *B. cenocepacia* (Krause et al., 2018). In this manner, macrophages carrying mutation F508del in CFTR also show a reduced association of LC3B with the BcCV (Abdulrahman et al., 2011). Importantly, intracellular *B. cenocepacia* decrease the transcription of Atg9b, Atg5, Atg12, and Atg8, suggesting that the downregulation of these autophagic components could be an additional strategy used by this bacterium to survive inside CF macrophages (**Figure 1**). In addition, in CF macrophages, the *Mirc1/Mir17-92* cluster works in a way similar to a negative regulator of autophagy. Thus, the downregulation of *Mir17* and *Mir20a* expression partially increases the clearance of *B. cenocepacia* by autophagy (Tazi et al., 2016). Therefore, the induction of autophagy with rapamycin on macrophages carrying mutation F508del in CFTR reduces the intracellular bacterial load and decrease the inflammation of the lungs of *B. cenocepacia*-infected mice F508del (Abdulrahman et al., 2011). Moreover, the autophagosome maturation in murine macrophages needs the expression of SQSTM1 (Komatsu et al., 2007). The depletion of SQSTM1 in F508del macrophages infected with *B. cenocepacia* results in the release of BECN1 from cytosolic CFTR aggregates with a consequent redistribution at BcCV in which LC3 is recruited to form functional autophagosomes (Abdulrahman et al., 2013). The pre-activation of macrophages either with IFN-γ or rapamycin increases the colocalization of SQSTM1 with BcCV to produce mature autophagosomes (Assani et al., 2014) (**Figure 1**). In addition, macrophages pre-activated with IFN-γ increase their ability to control intracellular *B. cenocepacia* to process and present bacterial antigens by class II MHC molecules to CD4 T-cells (Rosales-Reyes et al., 2020). Surprisingly, *B. cenocepacia* survives more efficiently in macrophages deficient in Gasdermin D (*gsdmd^-/-^*), an executioner of pyroptotic cell death. The deficiency of Gasdermin D is associated with a low rate of autophagosome formation (Estfanous et al., 2021).

In contrast, Al-khodor et al. report that *B. cenocepacia* strain J2315 in human monocyte-derived macrophages or mouse bone marrow-derived macrophages disrupt the membrane of the BcCV to escape into the cytosol, in which the bacterium is surrounded by actin, and recruits KDEL, ubiquitin, SQSTM1, and LC3B to form functional autophagosomes (Al-Khodor et al., 2014).

Altogether, the downregulation of autophagic pathway is a key strategy used by *B. cenocepacia* to survive and persist for long periods of time causing a severe inflammatory immune response that triggers lung deterioration in CF patients.

## Pseudomonas aeruginosa

*P. aeruginosa* is an environmentally ubiquitous Gram-negative bacterial pathogen that is associated with increased morbidity and mortality among CF patients (Govan and Harris, 1986). This bacterium colonizes the lower airways of CF-affected individuals. The ability of *P. aeruginosa* to survive in this microenvironment requires the efficient evasion of their recognition by the immune system. The downregulated expression of diverse virulence factors by constant acquisition of mutations in global regulator genes as the quorum sensing and the mismatch repair system are general mechanisms used by *P. aeruginosa* to mediate their adaptation and survival in this microenvironment (Rossi et al., 2020; Rosales-Reyes et al., 2021).

**Figure 2:**
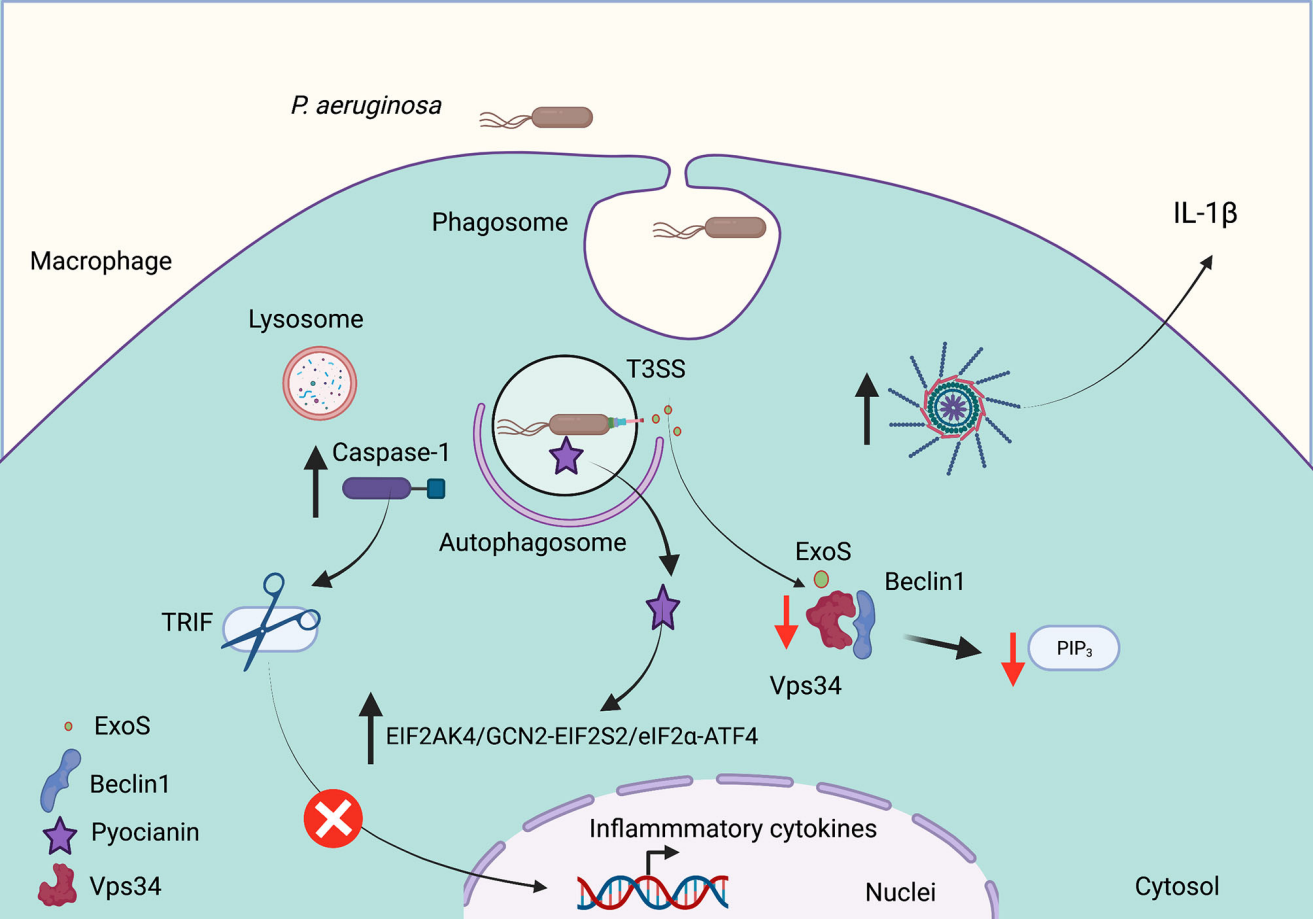
*Pseudomonas aeruginosa* subverts autophagy in cystic fibrosis. *P. aeruginosa* invades phagocytic and epithelial cells, which modulates the autophagic pathway to survive. Cell infection induces NLRP3 inflammasome activation to release IL-1b. Caspase-1 activation also mediates TRIF degradation to decrease the inflammatory response. The Type III Secretion System (T3SS) releases ExoS, a toxin with ADP ribosylation activity that decreases the Vsp34 activation to produce phosphatidylinositol (3,4,5) trisphosphate (PIP_3_). The release of pyocyanin promotes autophagy through the EIF2AK4/GCN2 (eukaryotic translation initiation factor 2 a kinase 4)–EIF2S2/eIF2α (eukaryotic translation initiation factor 2 subunit α)–ATF4 (activating transcription factor 4) pathway. Red arrows indicate decreased autophagy, and black arrows indicate increased autophagy. Created with BioRender.com.

Although *P. aeruginosa* was considered to be an extracellular opportunistic pathogen, it has been shown that it has the ability to gain access to phagocytic cells (Speert and Simpson, 1999). This bacterium induces autophagy in both macrophages and mast cells (Yuan et al., 2012; Junkins et al., 2013). In mouse and human macrophages, intracellular *P. aeruginosa* promotes autophagy to decrease phagocytosis and their intracellular bacterial killing (Wu et al., 2016). In these studies, the knockdown of ATG7 or Beclin1 increases both macrophage phagocytic activity as well as intracellular killing. Nevertheless, the autophagy induction by rapamycin decreases the expression of phagocytic receptors for *P. aeruginosa* (Wu et al., 2016). Additionally, in macrophages, *P. aeruginosa* induces the assembly and activation of the NLRP3 inflammasome; thus, active NLRP3 inflammasomes reduce the efficiency of macrophages to kill *P. aeruginosa* by the decreased formation of autophagosomes (**Figure 2**) (Deng et al., 2016). In addition, the inflammasome activation by *P. aeruginosa* does not require the type III secretion system. The inflammasome activation leads to TRIF processing by caspase-1 and decreases the NLRP3 inflammasome activation. Thus, inhibition of TRIF cleavage by caspase-1 increases the bactericidal activity mediated by autophagy (Jabir et al., 2014).

The ability of *P. aeruginosa* to modulate the formation of mature autophagosomes is a key strategy to ensure its survival in phagocytic cells. For example, Annexin A2, a member of the annexin family, interacts with Fam13A to activate the Rho GTPase to regulate the autophagosome formation after *P. aeruginosa* invasion through the Akt1-mTOR-ULK1/2 pathway (Li et al., 2015). *P. aeruginosa* produces pyocyanin (PYO), an important virulence factor required for their full virulence. PYO is a redox-active released pigment that interferes with several cellular functions in host cells including electron transport, gene expression, energy metabolism, cellular respiration, and an innate immune response (Rada and Leto, 2013). Deletion of the *phzM* gene (required for pyocyanin biosynthesis) significantly decreases autophagy induction. In addition, pyocyanin induces autophagy through the EIF2AK4/GCN2 (eukaryotic translation initiation factor 2 a kinase 4)–EIF2S2/eIF2α (eukaryotic translation initiation factor 2 subunit α)–ATF4 (activating transcription factor 4) pathway (**Figure 2**). The reduced pyocyanin production during chronic infections has been associated with better bacterial adaptation into the host (Yang et al., 2016). ExoS, a T3SS effector with the activity of ADP ribosylation, inhibits the host autophagy by decreasing the Vps34 kinase activity (Rao et al., 2021). Thus, the survival of *P. aeruginosa* inside phagocytic and epithelial cells requires a coordinated mechanism that ends in decreased autophagosome formation, leading to *P. aeruginosa* survival and persist for long periods of time, increasing the inflammatory immune response that gradually decreases lung function of individuals affected with CF.

## Autophagy in the Control of Intracellular Bacteria

*B. cenocepacia* and *P. aeruginosa* are two important opportunistic pathogens that produce chronic infection in CF lungs. Their ability to survive and persist into eukaryotic cells leads to the establishment of chronic inflammation and cell death. The bacterial survival in vacuoles suggests that the induction of autophagy could be an important strategy to destroy these pathogens that subvert autophagy. Rapamycin is a drug that induces autophagy, decreasing the intracellular load of *B. cenocepacia*. In the mouse model, Rapamycin also decreases lung inflammation induced by the *B. cenocepacia* infection (Abdulrahman et al., 2011). In addition, the treatment with gamma-interferon (IFNγ) also promotes the formation of autophagosomes, in which *B. cenocepacia* is destroyed (Assani et al., 2014). Thus, the intracellular processing of *B. cenocepacia* by pre-activated macrophages with IFNγ produces peptides that are presented by class II MHC molecules to CD4 T cells (Rosales-Reyes et al., 2020). Similar findings have been observed in macrophages infected with *P. aeruginosa* in which the treatment with rapamycin or IFNγ also induces autophagosomes in which the bacterium is destroyed (Yuan et al., 2012). These observations suggest that the induction of autophagy might decrease the intracellular survival of *B. cenocepacia* and *P. aeruginosa* to decrease the chronic colonization and inflammation of the CF lungs.

## Concluding Remarks

Eukaryotic cells can destroy intracellular microorganisms through the induction of autophagy. Autophagy is considered to be one of the first antimicrobial defense mechanisms used by several eukaryotic cells. This cellular process uses a distinct set of proteins that assemble a membrane around the vacuoles containing bacteria culminating in the destruction of the intracellular microorganisms. Bacterial antigens are processed in the autophagosome and the peptides generated are presented by class II MHC molecules to CD4 T cells to activate an adaptative immune response. Several microorganisms have evolved by developing strategies to evade autophagic degradation, allowing their survival and persistence. The bacterial persistence in the lungs of individuals affected with CF through the subversion of autophagy is a key factor that promotes chronic inflammation and decreases lung function, which ultimately compromises the life of affected individuals. The first bacterial pathogens associated with the colonization of the lower airways of newborn children with CF are non-typable *H. influenzae* and *S. aureus*. Progressively, these pathogens are replaced by *P. aeruginosa* and *B. cenocepacia* during adolescence and adulthood. The mechanisms described herein by which *B. cenocepacia* and *P. aeruginosa* subvert autophagy could help to establish better strategies to combat these intracellular pathogens that produce chronic infections in patients with CF.

## Author Contributions

RR-R conceived and supervised the review topics. VF-V, SV-R, and RR-R wrote the first draft. VF-V, SV-R, JL-F, RL, JS-P, and RR-R edited the manuscript. All authors contributed to the article and approved the submitted version.

## Funding

This work was supported by the Programa de Apoyo a Proyectos de Investigación e Innovación Tecnológica (No. IN224419 to RL), Universidad Nacional Autónoma de México.

## Conflict of Interest

The authors declare that the research was conducted in the absence of any commercial or financial relationships that could be construed as a potential conflict of interest.

## Publisher’s Note

All claims expressed in this article are solely those of the authors and do not necessarily represent those of their affiliated organizations, or those of the publisher, the editors and the reviewers. Any product that may be evaluated in this article, or claim that may be made by its manufacturer, is not guaranteed or endorsed by the publisher.

## References

[B1] Abdulrahman B. A. Khweek A. A. Akhter A. Caution K. Kotrange S. Abdelaziz D. H. A.. (2011). Autophagy Stimulation by Rapamycin Suppresses Lung Inflammation and Infection by Burkholderia Cenocepacia in a Model of Cystic Fibrosis. Autophagy 7, 1359–1370. doi: 10.4161/auto.7.11.17660 21997369 PMC3359483

[B2] Abdulrahman B. A. Khweek A. A. Akhter A. Caution K. Tazi M. Hassan H.. (2013). Depletion of the Ubiquitin-Binding Adaptor Molecule SQSTM1/p62 From Macrophages Harboring Cftr Δf508 Mutation Improves the Delivery of Burkholderia Cenocepacia to the Autophagic Machinery. J. Biol. Chem. 288, 2049–2058. doi: 10.1074/JBC.M112.411728 23148214 PMC3548511

[B3] Al-Khodor S. Marshall-Batty K. Nair V. Ding L. Greenberg D. E. Fraser I. D. C. (2014). *Burkholderia Cenocepacia* J2315 Escapes to the Cytosol and Actively Subverts Autophagy in Human Macrophages. Cell. Microbiol. 16, 378–395. doi: 10.1111/CMI.12223 24119232 PMC3927784

[B4] Assani K. Tazi M. F. Amer A. O. Kopp B. T. (2014). IFN-γ Stimulates Autophagy-Mediated Clearance of *Burkholderia Cenocepacia* in Human Cystic Fibrosis Macrophages. PloS One 9, e96681. doi: 10.1371/JOURNAL.PONE.0096681 24798083 PMC4010498

[B5] Bareil C. Bergougnoux A. (2020). CFTR Gene Variants, Epidemiology and Molecular Pathology. Arch. Pediatr. 1 Suppl(1), eS8–eS12. doi: 10.1016/S0929-693X(20)30044-0 32172939

[B6] Blanchard A. C. Waters V. J. (2019). Microbiology of Cystic Fibrosis Airway Disease. Semin. Respir. Crit. Care Med. 40, 727–736. doi: 10.1055/s-0039-1698464 31887768 PMC7117079

[B7] Boyle M. P. (2007). Adult Cystic Fibrosis. JAMA 298, 1787–1793. doi: 10.1001/jama.298.15.1787 17940235

[B8] Brockman S. M. Bodas M. Silverberg D. Sharma A. Vij N. (2017). Dendrimer-Based Selective Autophagy-Induction Rescues Δf508-CFTR and Inhibits Pseudomonas Aeruginosa Infection in Cystic Fibrosis. PloS One 12, e0184793. doi: 10.1371/journal.pone.0184793 28902888 PMC5597233

[B9] Burns J. L. Jonas M. Chi E. Y. Clark D. K. Berger A. Griffith A. (1996). Invasion of Respiratory Epithelial Cells by Burkholderia (*Pseudomonas*) *Cepacia* . Infect. Immunm 64, 4056–4059. doi: 10.1128/iai.64.10.4054-4059.1996 PMC174336 8926068

[B10] Cox M. J. Allgaier M. Taylor B. Baek M. S. Huang Y. J. Daly R. A.. (2010). Airway Microbiota and Pathogen Abundance in Age-Stratified Cystic Fibrosis Patients. PloS One 5, e11044. doi: 10.1371/journal.pone.0011044 20585638 PMC2890402

[B11] Deng Q. Wang Y. Zhang Y. Li M. Li D. Huang X.. (2016). *Pseudomonas Aeruginosa* Triggers Macrophage Autophagy To Escape Intracellular Killing by Activation of the NLRP3 Inflammasome. Infect. Immun. 84, 56–66. doi: 10.1128/IAI.00945-15 26467446 PMC4694000

[B12] De Stefano D. Villella V. R. Esposito S. Tosco A. Sepe A. De Gregorio F.. (2014). Restoration of CFTR Function in Patients With Cystic Fibrosis Carrying the F508del-CFTR Mutation. Autophagy 10, 2053–2074. doi: 10.4161/15548627.2014.973737 25350163 PMC4502695

[B13] Di A. Brown M. E. Deriy L. V. Li C. Szeto F. L. Chen Y.. (2006). CFTR Regulates Phagosome Acidification in Macrophages and Alters Bactericidal Activity. Nat. Cell Biol. 8, 933–944. doi: 10.1038/ncb1456 16921366

[B14] Dikic I. Elazar Z. (2018). Mechanism and Medical Implications of Mammalian Autophagy. Nat. Rev. Mol. Cell Biol. 19, 349–364. doi: 10.1038/s41580-018-0003-4 29618831

[B15] Estfanous S. Krause K. Anne M. N. K. Eltobgy M. Caution K. Khweek A. A.. (2021). Gasdermin D Restricts Burkholderia Cenocepacia Infection *In Vitro* and *In Vivo* . Sci. Rep. 111 11, 1–20. doi: 10.1038/s41598-020-79201-5 PMC7807041 33441602

[B16] Ferrari E. Monzani R. Villella V. R. Esposito S. Saluzzo F. Rossin F.. (2017). Cysteamine Re-Establishes the Clearance of Pseudomonas Aeruginosa by Macrophages Bearing the Cystic Fibrosis-Relevant F508del-CFTR Mutation. Cell Death Dis. 8, e2544. doi: 10.1038/cddis.2016.476 28079883 PMC5386380

[B17] Gilardini Montani M. S. Santarelli R. Granato M. Gonnella R. Torrisi M. R. Faggioni A.. (2019). EBV Reduces Autophagy, Intracellular ROS and Mitochondria to Impair Monocyte Survival and Differentiation. Autophagy 15, 652–667. doi: 10.1080/15548627.2018.1536530 30324853 PMC6526866

[B18] Govan J. R. Harris G. S. (1986). *Pseudomonas Aeruginosa* and Cystic Fibrosis: Unusual Bacterial Adaptation and Pathogenesis. Microbiol. Sci. 3, 302–308. 3155268

[B19] Harris J. Lang T. Thomas J. P. W. Sukkar M. B. Nabar N. R. Kehrl J. H. (2017). Autophagy and Inflammasomes. Mol. Immunol. 86, 10–15. doi: 10.1016/j.molimm.2017.02.013 28249679

[B20] Huang J. Brumell J. H. (2014). Bacteria-Autophagy Interplay: A Battle for Survival. Nat. Rev. Microbiol. 12, 101–114. doi: 10.1038/nrmicro3160 24384599 PMC7097477

[B21] Isles A. Maclusky I. Corey M. Gold R. Prober C. Fleming P.. (1984). *Pseudomonas Cepacia* Infection in Cystic Fibrosis: An Emerging Problem. J. Pediatr. 104, 206–210. doi: 10.1016/S0022-3476(84)80993-2 6420530

[B22] Jabir M. S. Ritchie N. D. Li D. Bayes H. K. Tourlomousis P. Puleston D.. (2014). Caspase-1 Cleavage of the TLR Adaptor TRIF Inhibits Autophagy and B-Interferon Production During Pseudomonas Aeruginosa Infection. Cell Host Microbe 15, 214–227. doi: 10.1016/j.chom.2014.01.010 24528867 PMC7617833

[B23] Jackson A. D. Goss C. H. (2018). Epidemiology of CF: How Registries can be Used to Advance Our Understanding of the CF Population. J. Cyst. Fibros. 17, 297–305. doi: 10.1016/j.jcf.2017.11.013 29275954

[B24] Junkins R. D. Shen A. Rosen K. McCormick C. Lin T.-J. (2013). Autophagy Enhances Bacterial Clearance During P. Aeruginosa Lung Infection. PloS One 8, e72263. doi: 10.1371/journal.pone.0072263 24015228 PMC3756076

[B25] Komatsu M. Waguri S. Koike M. Sou Y. Ueno T. Hara T.. (2007). Homeostatic Levels of P62 Control Cytoplasmic Inclusion Body Formation in Autophagy-Deficient Mice. Cell 131, 1149–1163. doi: 10.1016/J.CELL.2007.10.035 18083104

[B26] Kopp B. T. Abdulrahman B. A. Khweek A. A. Kumar S. B. Akhter A. Montione R.. (2012). Exaggerated Inflammatory Responses Mediated by Burkholderia Cenocepacia in Human Macrophages Derived From Cystic Fibrosis Patients. Biochem. Biophys. Res. Commun. 424, 221–227. doi: 10.1016/j.bbrc.2012.06.066 22728038 PMC3408781

[B27] Krause K. Caution K. Badr A. Hamilton K. Saleh A. Patel K.. (2018). CASP4/caspase-11 Promotes Autophagosome Formation in Response to Bacterial Infection. Autophagy 14, 1928–1942. doi: 10.1080/15548627.2018.1491494 30165781 PMC6152495

[B28] Lamothe J. Huynh K. K. Grinstein S. Valvano M. A. (2007). Intracellular Survival of *Burkholderia Cenocepacia* in Macrophages is Associated With a Delay in the Maturation of Bacteria-Containing Vacuoles. Cell. Microbiol. 9, 40–53. doi: 10.1111/j.1462-5822.2006.00766.x 16869828

[B29] Lamothe J. Valvano M. A. (2008). *Burkholderia Cenocepacia*-Induced Delay of Acidification and Phagolysosomal Fusion in Cystic Fibrosis Transmembrane Conductance Regulator (CFTR)-Defective Macrophages. Microbiology 154, 3825–3834. doi: 10.1099/mic.0.2008/023200-0 19047750

[B30] Li R. Tan S. Yu M. Jundt M. C. Zhang S. Wu M. (2015). Annexin A2 Regulates Autophagy in Pseudomonas Aeruginosa Infection Through the Akt1–mTOR–ULK1/2 Signaling Pathway. J. Immunol. 195, 3901 – 3911. doi: 10.4049/jimmunol.1500967 26371245 PMC4592832

[B31] Luciani A. Villella V. R. Esposito S. Brunetti-Pierri N. Medina D. Settembre C.. (2010). Defective CFTR Induces Aggresome Formation and Lung Inflammation in Cystic Fibrosis Through ROS-Mediated Autophagy Inhibition. Nat. Cell Biol. 12, 863–875. doi: 10.1038/ncb2090 20711182

[B32] Mahenthiralingam E. Urban T. A. Goldberg J. B. (2005). The Multifarious, Multireplicon *Burkholderia Cepacia* Complex. Nat. Rev. Microbiol. 3, 144–156. doi: 10.1038/nrmicro1085 15643431

[B33] Martin D. W. Mohr C. D. (2000). Invasion and Intracellular Survival of *Burkholderia Cepacia* . Infect. Immun. 68, 24–29. doi: 10.1128/IAI.68.1.24-29.2000 10603364 PMC97097

[B34] Nezis I. P. Simonsen A. Sagona A. P. Finley K. Gaumer S. Contamine D.. (2008). Ref(2)P, the Drosophila Melanogaster Homologue of Mammalian P62, is Required for the Formation of Protein Aggregates in Adult Brain. J. Cell Biol. 180, 1065–1071. doi: 10.1083/jcb.200711108 18347073 PMC2290837

[B35] O’Sullivan B. P. Freedman S. D. (2009). Cystic Fibrosis. Lancet 373, 1891–1904. doi: 10.1016/S0140-6736(09)60327-5 19403164

[B36] Painter R. G. Valentine V. G. Lanson N. A. J. Leidal K. Zhang Q. Lombard G.. (2006). CFTR Expression in Human Neutrophils and the Phagolysosomal Chlorination Defect in Cystic Fibrosis. Biochemistry 45, 10260–10269. doi: 10.1021/bi060490t 16922501 PMC2931333

[B37] Porto P. Cifani N. Guarnieri S. Di Domenico E. G. Mariggiò M. A. Spadaro F.. (2011). Dysfunctional CFTR Alters the Bactericidal Activity of Human Macrophages Against Pseudomonas Aeruginosa. PloS One 6, e19970. doi: 10.1371/journal.pone.0019970 21625641 PMC3097223

[B38] Rada B. Leto T. L. (2013). Pyocyanin Effects on Respiratory Epithelium: Relevance in *Pseudomonas Aeruginosa* Airway Infections. Trends Microbiol. 21, 73–81. doi: 10.1016/j.tim.2012.10.004 23140890 PMC3565070

[B39] Rao L. de la Rosa I. Xu Y. Sha Y. Bhattacharya A. Holtzman M. J.. (2021). *Pseudomonas Aeruginosa* Survives in Epithelia by ExoS-Mediated Inhibition of Autophagy and mTOR. EMBO Rep. 22, e50613. doi: 10.15252/embr.202050613 33345425 PMC7857434

[B40] Rosales-Reyes R. Aubert D. F. Tolman J. S. Amer A. O. Valvano M. A. (2012a). Burkholderia Cenocepacia Type VI Secretion System Mediates Escape of Type II Secreted Proteins Into the Cytoplasm of Infected Macrophages. PloS One 7, 1–14. doi: 10.1371/journal.pone.0041726 PMC3405007 22848580

[B41] Rosales-Reyes R. Garza-Villafuerte P. Vences-Vences D. Aubert D. F. Aca-Teutle R. Ortiz-Navarrete V. F.. (2020). Interferon-Gamma-Activated Macrophages Infected With Burkholderia Cenocepacia Process and Present Bacterial Antigens to T-Cells by Class I and II Major Histocompatibility Complex Molecules. Emerg. Microbes Infect. 9, 2000–2012. doi: 10.1080/22221751.2020.1818632 32873215 PMC7534305

[B42] Rosales-Reyes R. Skeldon A. M. Aubert D. F. Valvano M. A. (2012b). The Type VI Secretion System of Burkholderia Cenocepacia Affects Multiple Rho Family GTPases Disrupting the Actin Cytoskeleton and the Assembly of NADPH Oxidase Complex in Macrophages. Cell. Microbiol. 14, 255–273. doi: 10.1111/j.1462-5822.2011.01716.x 22023353

[B43] Rosales-Reyes R. Vargas-Roldán S. Y. Lezana-Fernández J. L. Santos-Preciado J. I. (2021). *Pseudomonas Aeruginosa*: Genetic Adaptation, A Strategy for its Persistence in Cystic Fibrosis. Arch. Med. Res. 52, 357–361. doi: 10.1016/j.arcmed.2020.12.004 33309309

[B44] Rossi E. La Rosa R. Bartell J. A. Marvig R. L. Haagensen J. A. J. Sommer L. M.. (2020). *Pseudomonas Aeruginosa* Adaptation and Evolution in Patients With Cystic Fibrosis. Nat. Rev. Microbiol. 19, 331–342. doi: 10.1038/s41579-020-00477-5 33214718

[B45] Saini L. S. Galsworthy S. B. John M. A. Valvano M. A. (1999). Intracellular Survival of *Burkholderia Cepacia* Complex Isolates in the Presence of Macrophage Cell Activation. Microbiology 145, 3465–3475. doi: 10.1099/00221287-145-12-3465 10627044

[B46] Sajjan U. S. Yang J. H. Hershenson M. B. LiPuma J. J. (2006). Intracellular Trafficking and Replication of *Burkholderia Cenocepacia* in Human Cystic Fibrosis Airway Epithelial Cells. Cell. Microbiol. 8, 1456–1466. doi: 10.1111/J.1462-5822.2006.00724.X 16922864

[B47] Scoffone V. C. Chiarelli L. R. Trespidi G. Mentasti M. Riccardi G. Buroni S. (2017). Burkholderia Cenocepacia Infections in Cystic Fibrosis Patients: Drug Resistance and Therapeutic Approaches. Front. Microbiol. 8, 1592. doi: 10.3389/fmicb.2017.01592 28878751 PMC5572248

[B48] Shteinberg M. Haq I. J. Polineni D. Davies J. C. (2021). Cystic Fibrosis. Lancet (London England) 397, 2195–2211. doi: 10.1016/S0140-6736(20)32542-3 34090606

[B49] Smith J. J. Travis S. M. Greenberg E. P. Welsh M. J. (1996). Cystic Fibrosis Airway Epithelia Fail to Kill Bacteria Because of Abnormal Airway Surface Fluid. Cell 85, 229–236. doi: 10.1016/s0092-8674(00)81099-5 8612275

[B50] Speert D. P. Simpson D. A. (1999). “Phagocytosis of Pseudomonas Aeruginosa,” in Phagocytosis: Microbial Invasion. Eds. S. B. T.-A. @ in C. and M. B. @ of M Gordon O. (JAI), Amsterdam, Netherlands: Elsevier. pp. 159–172. doi: 10.1016/S1874-5172(99)80010-8

[B51] Tablan O. C. Chorba T. L. Schidlow D. V. White J. W. Hardy K. A. Gilligan P. H.. (1985). *Pseudomonas Cepacia* Colonization in Patients With Cystic Fibrosis: Risk Factors and Clinical Outcome. J. Pediatr. 107, 382–387. doi: 10.1016/S0022-3476(85)80511-4 4032134

[B52] Tazi M. F. Dakhlallah D. A. Caution K. Gerber M. M. Chang S.-W. Khalil H.. (2016). Elevated Mirc1/Mir17-92 Cluster Expression Negatively Regulates Autophagy and CFTR (Cystic Fibrosis Transmembrane Conductance Regulator) Function in CF Macrophages. Autophagy 12, 2026–2037. doi: 10.1080/15548627.2016.1217370 27541364 PMC5103351

[B53] Tsui L. C. Buchwald M. Barker D. Braman J. C. Knowlton R. Schumm J. W.. (1985). Cystic Fibrosis Locus Defined by a Genetically Linked Polymorphic DNA Marker. Science 230, 1054–1057. doi: 10.1126/science.2997931 2997931

[B54] Villella V. R. Esposito S. Bruscia E. M. Vicinanza M. Cenci S. Guido S.. (2013). Disease-Relevant Proteostasis Regulation of Cystic Fibrosis Transmembrane Conductance Regulator. Cell Death Differ. 20, 1101–1115. doi: 10.1038/cdd.2013.46 23686137 PMC3705602

[B55] Wu Y. Li D. Wang Y. Chen K. Yang K. Huang X.. (2016). *Pseudomonas Aeruginosa* Promotes Autophagy to Suppress Macrophage-Mediated Bacterial Eradication. Int. Immunopharmacol. 38, 214–222. doi: 10.1016/j.intimp.2016.04.044 27295610

[B56] Yang Z.-S. Ma L.-Q. Zhu K. Yan J.-Y. Bian L. Zhang K.-Q.. (2016). *Pseudomonas* Toxin Pyocyanin Triggers Autophagy: Implications for Pathoadaptive Mutations. Autophagy 12, 1015–1028. doi: 10.1080/15548627.2016.1170256 27159636 PMC4922443

[B57] Yoshimura K. Nakamura H. Trapnell B. C. Chu C. S. Dalemans W. Pavirani A.. (1991). Expression of the Cystic Fibrosis Transmembrane Conductance Regulator Gene in Cells of non-Epithelial Origin. Nucleic Acids Res. 19, 5417–5423. doi: 10.1093/nar/19.19.5417 1717947 PMC328907

[B58] Yuan K. Huang C. Fox J. Laturnus D. Carlson E. Zhang B.. (2012). Autophagy Plays an Essential Role in the Clearance of Pseudomonas Aeruginosa by Alveolar Macrophages. J. Cell Sci. 125, 507–515. doi: 10.1242/jcs.094573 22302984 PMC3283879

